# Genetic inactivation of *ANGPTL4* improves glucose homeostasis and is associated with reduced risk of diabetes

**DOI:** 10.1038/s41467-018-04611-z

**Published:** 2018-06-13

**Authors:** Viktoria Gusarova, Colm O’Dushlaine, Tanya M. Teslovich, Peter N. Benotti, Tooraj Mirshahi, Omri Gottesman, Cristopher V. Van Hout, Michael F. Murray, Anubha Mahajan, Jonas B. Nielsen, Lars Fritsche, Anders Berg Wulff, Daniel F. Gudbjartsson, Marketa Sjögren, Connor A. Emdin, Robert A. Scott, Wen-Jane Lee, Aeron Small, Lydia C. Kwee, Om Prakash Dwivedi, Rashmi B. Prasad, Shannon Bruse, Alexander E. Lopez, John Penn, Anthony Marcketta, Joseph B. Leader, Christopher D. Still, H. Lester Kirchner, Uyenlinh L. Mirshahi, Amr H. Wardeh, Cassandra M. Hartle, Lukas Habegger, Samantha N. Fetterolf, Teresa Tusie-Luna, Andrew P. Morris, Hilma Holm, Valgerdur Steinthorsdottir, Patrick Sulem, Unnur Thorsteinsdottir, Jerome I. Rotter, Lee-Ming Chuang, Scott Damrauer, David Birtwell, Chad M. Brummett, Amit V. Khera, Pradeep Natarajan, Marju Orho-Melander, Jason Flannick, Luca A. Lotta, Cristen J. Willer, Oddgeir L. Holmen, Marylyn D. Ritchie, David H. Ledbetter, Andrew J. Murphy, Ingrid B. Borecki, Jeffrey G. Reid, John D. Overton, Ola Hansson, Leif Groop, Svati H. Shah, William E. Kraus, Daniel J. Rader, Yii-Der I. Chen, Kristian Hveem, Nicholas J. Wareham, Sekar Kathiresan, Olle Melander, Kari Stefansson, Børge G. Nordestgaard, Anne Tybjærg-Hansen, Goncalo R. Abecasis, David Altshuler, Jose C. Florez, Michael Boehnke, Mark I. McCarthy, George D. Yancopoulos, David J. Carey, Alan R. Shuldiner, Aris Baras, Frederick E. Dewey, Jesper Gromada

**Affiliations:** 1 Regeneron Pharmaceuticals, Tarrytown, 10591 NY USA; 2 Regeneron Genetics Center, Tarrytown, 10591 NY USA; 3 Geisinger, Danville, 17822 PA USA; 4 Wellcome Centre for Human Genetics, University of Oxford, Oxford, OX3 7BN UK; 5 Department of Internal Medicine, Division of Cardiovascular Medicine, University of Michigan, University of Michigan, Ann Arbor, 48109 MI USA; 6 Department of Human Genetics, University of Michigan, University of Michigan, Ann Arbor, 48109 MI USA; 7 Department of Biostatistics and Center for Statistical Genetics, University of Michigan, Ann Arbor, 48109 MI USA; 8 Department of Clinical Biochemistry, Rigshospitalet, Copenhagen University Hospital, Copenhagen, 2100 Denmark; 9 deCODE Genetics/Amgen, Inc., Reykjavik, 101 Iceland; 10 Department of Clinical Sciences, Malmö, Lund University, Malmö, 221 Sweden; 11 Program in Medical and Population Genetics, Broad Institute, Cambridge, 02142 MA USA; 12 MRC Epidemiology Unit, Institute of Metabolic Science, University of Cambridge School of Clinical Medicine, Cambridge Biomedical Campus, Addenbrooke’s Hospital, Cambridge, CB2 0QQ UK; 13 Department of Medical Research, Taichung Veterans General Hospital, Taichung, 40705 Taiwan; 14 Department of Social Work, Tunghai University, Taichung, 40704 Taiwan; 15 Department of Genetics, Perelman School of Medicine, University of Pennsylvania, Philadelphia, 19104 PA USA; 16 Department of Medicine, Perelman School of Medicine, University of Pennsylvania, Philadelphia, 19104 USA; 17 Division of Cardiology, Department of Medicine; Molecular Physiology Institute, School of Medicine, Duke University, Durham, 27710 NC USA; 18 Finnish Institute of Molecular Medicine (FIMM), Helsinki University, Helsinki, 00170 Finland; 19 Department of Clinical Sciences, Clinical Research Centre, Lund University, Malmö, 221 Sweden; 20 Instituto de Investigaciones Biomédicas, UNAM, Coyoacán, 04510 Mexico City, Mexico; 21 Unidad de Biología Molecular y Medicina Genómica, UNAM/INCMNSZ Instituto Nacional de Ciencias Médicas y Nutrición Salvador Zubirán, Mexico City, 14080 Mexico; 22 Department of Biostatistics, University of Liverpool, Liverpool, L69 7ZX UK; 23 Estonian Genome Center, University of Tartu, Tartu, 50090 Estonia; 24 Institute for Translational Genomics and Population Sciences, Departments of Pediatrics and Medicine, LABioMed at Harbor-UCLA Medical Center, Torrance, 90502 CA USA; 25 Division of Endocrinology & Metabolism, Department of Internal Medicine, National Taiwan University Hospital, Taipei, 10617 Taiwan; 26 Institute of Preventive Medicine, School of Public Health, National Taiwan University, Taipei, 10617 Taiwan; 27 Department of Surgery, Perelman School of Medicine, University of Pennsylvania, Philadelphia, 19104 PA USA; 28 Department of Surgery, Corporal Michael Crescenz VA Medical Center, Philadelphia, 19104 PA USA; 29 Department of Anesthesiology, University of Michigan, Ann Arbor, 48109 MI USA; 30 Center for Human Genetic Research, Cardiovascular Research Center and Cardiology Division, Massachusetts General Hospital, Harvard Medical School, Boston, 02114 MA USA; 31 Center for Human Genetic Research, Massachusetts General Hospital, Harvard Medical School, Boston, 02114 MA USA; 32 Department of Computational Medicine and Bioinformatics, University of Michigan, Ann Arbor, 48109 MI USA; 33 HUNT Research Centre, Department of Public Health and General Practice, Norwegian University of Science and Technology, Levanger, 7601 Norway; 34 K.G. Jebsen Center for Genetic Epidemiology, Department of Public Health, Norwegian University of Science and Technology, Trondheim, 7491 Norway; 35 Department of Medicine, Levanger Hospital, Nord-Trøndelag Health Trust, Levanger, 7601 Norway; 36 The Copenhagen General Population Study, Herlev and Gentofte Hospital, Copenhagen University Hospital, Copenhagen, 2730 Denmark; 37 Department of Clinical Biochemistry, Herlev and Gentofte Hospital, Copenhagen University Hospital, Copenhagen, 2730 Denmark; 38 The Copenhagen City Heart Study, Frederiksberg Hospital, Copenhagen University Hospital, Copenhagen, 2400 Denmark; 39 Faculty of Health and Medical Sciences, University of Copenhagen, Copenhagen, 2200 Denmark; 40 Department of Molecular Biology, Diabetes Unit, and Center for Human Genetic Research, Massachusetts General Hospital, Boston, 02114 MA USA; 41 Departments of Genetics and Medicine, Harvard Medical School, Boston, 02115 MA USA; 42 Department of Biology, Massachusetts Institute of Technology, Cambridge, 02139 MA USA; 43 Diabetes Unit and Center for Human Genetic Research, Massachusetts General Hospital, Boston, 02115 MA USA; 44 Programs in Metabolism and Medical & Population Genetics, Broad Institute, Cambridge, 02142 MA USA; 45 Department of Medicine, Harvard Medical School, Boston, 02115 MA USA; 46 Oxford Centre for Diabetes, Endocrinology and Metabolism, University of Oxford, Churchill Hospital, Oxford, OX3 7LE UK; 47 Oxford NIHR Biomedical Research Centre, Churchill Hospital, Oxford, OX4 2PG UK

## Abstract

Angiopoietin-like 4 (ANGPTL4) is an endogenous inhibitor of lipoprotein lipase that modulates lipid levels, coronary atherosclerosis risk, and nutrient partitioning. We hypothesize that loss of ANGPTL4 function might improve glucose homeostasis and decrease risk of type 2 diabetes (T2D). We investigate protein-altering variants in *ANGPTL4* among 58,124 participants in the DiscovEHR human genetics study, with follow-up studies in 82,766 T2D cases and 498,761 controls. Carriers of p.E40K, a variant that abolishes ANGPTL4 ability to inhibit lipoprotein lipase, have lower odds of T2D (odds ratio 0.89, 95% confidence interval 0.85–0.92, *p* = 6.3 × 10^−10^), lower fasting glucose, and greater insulin sensitivity. Predicted loss-of-function variants are associated with lower odds of T2D among 32,015 cases and 84,006 controls (odds ratio 0.71, 95% confidence interval 0.49–0.99, *p* = 0.041). Functional studies in *Angptl4*-deficient mice confirm improved insulin sensitivity and glucose homeostasis. In conclusion, genetic inactivation of ANGPTL4 is associated with improved glucose homeostasis and reduced risk of T2D.

## Abstract (web summary)

Genetic variation in *ANGPTL4* is associated with lipid traits. Here, the authors find that predicted loss-of-function variants in *ANGPTL4* are associated with glucose homeostasis and reduced risk of type 2 diabetes and that *Angptl4*^−*/*−^ mice on a high-fat diet show improved insulin sensitivity.

## Introduction

The lipoprotein lipase (LPL) pathway has emerged as an attractive therapeutic target for reducing lipid levels and cardiovascular risk. Angiopoietin-like 4 (ANGPTL4) is a widely expressed endogenous inhibitor of LPL that modulates free fatty acid delivery to adipose and oxidative tissues during fasting and fed states^1–4^. Genetic variants in *ANGPTL4* are robustly associated with triglyceride and high-density lipoprotein cholesterol (HDL-C) levels in humans^5,6^, an observation which is supported by reduction in triglyceride and increase in HDL-C levels in response to antibody inhibition of ANGPTL4 in animal models^7,8^. Furthermore, inactivating variants in *ANGPTL4* are associated with reduced risk of coronary artery disease in humans, suggesting that ANGPTL4 and related LPL modulators may be targets for modification of dyslipidemia-related atherosclerotic cardiovascular disease^8,9^. Because modulation of LPL activity in oxidative tissues affects free fatty acid delivery^10–13^, and thereby nutrient partitioning and insulin sensitivity, endogenous regulators of LPL activity may impact glucose homeostasis and risk for development of type 2 diabetes. Together, these observations suggest that genetic inhibition of ANGPTL4 function might have a favorable impact on glucose homeostasis in humans and reduce risk of type 2 diabetes. This hypothesis is supported by a recent report of reduced type 2 diabetes risk associated with the p.E40K variant that abolishes ANGPTL4 ability to inhibit LPL^14,15^. It is not yet known whether p.E40K affects glucose homeostasis in non-diabetics, whether other variants that abolish ANGPTL4 function modify type 2 diabetes risk, or how loss of ANGPTL4 function modifies glucose homeostasis and type 2 diabetes risk.

There are conflicting published reports of the relationship between ANGPTL4 function and glucose homeostasis in animal models. ANGPTL4 overexpression in mice has been variously reported to have no effect on blood glucose levels^4,16^, to decrease blood glucose and improve glucose tolerance^17,18^, and to impair glucose tolerance^19^. The discrepancies in these results may be related to the level and the site of ANGPTL4 overexpression. Metabolic investigations in whole body *Angptl4*-deficient mice may inform on the glycemic effects of global ANGPTL4 inhibition.

In this study, we examine the association of genetic variants that abolish ANGPTL4 function with fasting glucose, oral glucose tolerance, and risk for type 2 diabetes in 58,124 individuals of European ancestry sampled from a large US health care population, and in 13 additional datasets comprising 82,766 type 2 diabetes cases and 498,761 controls. We also evaluate the functional consequences of rare and novel genetic variants identified by exome sequencing, as well as the effect of *Angptl4* deletion on insulin sensitivity and glucose homeostasis.

We find that p.E40K in *ANGPTL4* is associated with lower fasting glucose and greater insulin sensitivity in humans, as well as reduced risk of type 2 diabetes. Other rare predicted loss-of-function variants in *ANGPTL4* are also associated with lower risk of type 2 diabetes, providing additional allelic evidence that genetic loss of ANGPTL4 function improves glucose homeostasis in humans. *Angptl4*-deficient mice manifest greater insulin sensitivity and improved glucose homeostasis, further supporting for the conclusion that genetic inactivation of ANGPTL4 improves glucose homeostasis and reduces risk of type 2 diabetes.

## Results

### Whole exome sequencing identifies rare variants in *ANGPTL4*

Whole exome sequencing was performed in 58,124 adult participants of European ancestry in the DiscovEHR study. Demographics and clinical characteristics of the study population are shown in Supplementary Table 1. We identified 2235 heterozygotes and 26 homozygotes for p.E40K (minor allele frequency (MAF) 1.97%), which has been shown to abolish ANGPTL4 ability to inhibit LPL^15^. Twenty-one additional rare predicted loss-of-function (pLoF) variants in *ANGPTL4* were identified: 8 premature stop variants, 10 open reading frame shifting insertion/deletion variants, 2 splice acceptor variants, and 1 splice donor variant (Supplementary Table 2). In all, 125 individuals were heterozygous for these pLoF variants (cumulative allele frequency 0.11%). The most frequently observed pLoF variant was a single-nucleotide deletion at Glycine 313 (p.G313fs), which was observed in 69 DiscovEHR participants. We did not observe homozygotes or compound heterozygotes for these genetic variants in the DiscovEHR population.

### *ANGPTL4* p.E40K reduces the risk of type 2 diabetes

**Fig. 1:**
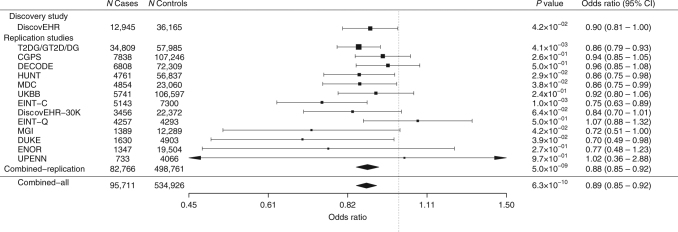
*ANGPTL4* p.E40K associates with reduced risk of type 2 diabetes. The association between the p.E40K variant and type 2 diabetes was tested in each study using logistic or Firth logistic regression, coding genotypes according to an additive model. “Combined” effects were calculated using inverse variance weighted fixed-effects meta-analysis. For each study, the squares indicate the odds ratio and lines indicate 95% confidence intervals. The square size is proportional to the standard error of the estimate. CI confidence interval, CGPS Copenhagen General Population Studies, DECODE deCODE, DiscovEHR DiscovEHR Discovery Study, DiscovEHR DiscovEHR-30K, DiscovEHR 30K Replication Study, EINT-C EPIC interact–CoreExome, EINT-Q EPIC Interact–Quad660, ENOR EPIC Norfolk, HUNT the Nord-Trøndelag Health study, MDC Malmo Diet and Cancer Study, MGI the Michigan Genomics Initiative, TD2G/GT2D/DG combined analysis of T2D-GENES, GoT2D, and DIAGRAM studies, UKBB United Kingdom Biobank. The study populations are described in full in Supplementary Table 4 and in the Supplementary Note

We examined associations of *ANGPTL4* p.E40K with type 2 diabetes defined by an Electronic Health Record (EHR) algorithm in the DiscovEHR study (Fig. 1). The allele frequency of p.E40K was lower in type 2 diabetes cases (1.82%: 461 heterozygotes and 5 homozygotes among 12,945 cases) than in non-diabetic controls (2.07%: 1457 heterozygotes and 19 homozygotes among 36,165 controls), corresponding to a reduction in odds of diabetes of 10% after adjusting for age, age^2^, sex, and four principal components of ancestry (odds ratio ((OR) 0.90, 95% confidence interval (CI) 0.81–1.00, *p* = 0.042). Type 2 diabetes cases and non-diabetic controls were not selected by age in DiscovEHR, and, as expected, type 2 diabetes cases were older than non-diabetic controls. However, there was not a meaningful difference in age between genotype groups, suggesting that age was not likely a confounder of this analysis (Supplementary Table 3), and the association of p.E40K with type 2 diabetes persisted after adjusting for linear and quadratic effects of age. Adjustment for body mass index (BMI) did not meaningfully change the association with type 2 diabetes (OR 0.85, 95% CI 0.76–0.96, *p* = 0.0076). We sought replication of this observation of reduced odds of type 2 diabetes among p.E40K variant carriers in 13 additional studies comprising 82,766 type 2 diabetes cases and 498,761 controls (Supplementary Table 4 and Fig. 1). Inverse variance weighted fixed-effects meta-analysis of the association of p.E40K with type 2 diabetes in these cohorts yielded an overall OR for type 2 diabetes of 0.88 (95% CI 0.85–0.92, *p* = 5.0 × 10^−9^). Inclusion of the DiscovEHR population in this meta-analysis yielded an OR of 0.89 for type 2 diabetes (95% CI 0.85–0.92, *p* = 6.3 × 10^−10^).

### *ANGPTL4* p.E40K improves glucose homeostasis

We also evaluated associations of p.E40K with fasting glucose in non-diabetic participants in the DiscovEHR study (*n* = 26,644 participants with fasting glucose measurements), an independent DiscovEHR replication cohort (*n* = 13,732), the UPenn study (*n* = 5782), the T2D-GENES, GoT2D, and DIAGRAM studies (*n* = 33,245), the Malmo Diet and Cancer Study (*n* = 4848), the HUNT study (*n* = 2714), and the deCODE study (n = 39,700); results are summarized in Supplementary Table 5. In five of these six studies, p.E40K was associated with lower fasting glucose, and nominally significant association with fasting glucose was observed in meta-analysis of these six studies (*p* = 0.0024, total *n* = 120,050). To further understand the association of p.E40K with glucose homeostasis in humans, we performed association analyses of the p.E40K variant with tolerance to a 75 g oral glucose challenge in up to 8081 non-diabetic participants in the NIDDM Botnia^20^ and Prevalence, Prediction, and Prevention of Diabetes-Botnia^21^ studies (Supplementary Table 6). In these studies, p.E40K was associated with nominally significantly lower insulin levels at 30 and 120 min after glucose administration, as well as lower insulinogenic index (β = −0.15 *Z* score units, standard error (SE) 0.047, *p* = 0.002) and higher insulin sensitivity index (β = 0.13 *Z* score units, SE 0.043, *p* = 0.0026). These findings suggest that p.E40K influences glucose homeostasis in non-diabetics via increased insulin sensitivity, and provide mechanistic insight into the association of p.E40K with reduced risk of type 2 diabetes.

### Rare pLoFs in ANGPTL4 reduce the risk of type 2 diabetes

To understand whether *ANGPTL4* pLoF variants were also associated with type 2 diabetes, we evaluated the prevalence of pLoFs in *ANGPTL4* in exome sequence data from 32,015 type 2 diabetes cases and 84,006 controls in six population case–control studies (Supplementary Table 7). In five of the six studies, *ANGTPL4* pLoF variants were less frequent among cases than controls (Table 1). We used a two-sided exact conditional test to combine the counts across studies given the low number of type 2 diabetes cases co-occurring with *ANGPTL4* pLoFs, finding 29% lower odds of type 2 diabetes among carriers of pLoF variants (OR 0.71, 95% CI 0.49–0.99, *p* = 0.041). Collectively, these findings indicate that genetic variants that abolish ANGPTL4 function are associated with improved insulin sensitivity and glucose homeostasis and reduced risk of type 2 diabetes in humans.

**Table 1:** Associations of *ANGPTL4* loss-of- function variants and type 2 diabetes

	*ANGPTL4* LoF carriers		Total		Frequency	
Study	Cases	Controls	Cases	Controls	Cases	Controls
DiscovEHR	22	85	12,969	36,217	0.0008	0.0012
DiscovEHR 30K replication	5	47	3,456	22,372	0.0007	0.0011
UPenn	3	13	734	4,066	0.0020	0.0016
Duke	2	10	1,630	4,903	0.0006	0.0010
TAICHI	0	3	4,392	4,699	0.0000	0.0003
DHS-EA	0	5	104	1,255	0.0000	0.0020
DHS-AA	0	1	357	2,028	0.0000	0.0002
T2D-Genes/GoT2D/DIAGRAM	14	19	8,373	8,466	0.0008	0.0011
Total	46	183	32,015	84,006	0.0007	0.0011

*AA* African American, *CI* confidence interval, *Duke* Duke CATHGEN cohort, *EA* European American, *TD2-Genes/GoT2D/DIAGRAM* combined analysis of T2D-GENES, GoT2D and DIAGRAM studies, *pLoF* predicted loss-of-function variant, *Penn* University of Pennsylvania Medicine Biobank, *TAICHI* TAIwan MetaboCHIp consortium

The overall odds ratio for *ANGPTL4* pLoFs and type 2 diabetes risk, using a two-sided exact conditional test, was 0.71 (95% CI 0.49–0.99, *p* = 0.041)

### *ANGPTL4* p.E40K primarily influences metabolic measures

Previous studies have noted a potential toxicity, abdominal lymphadenopathy, in *Angptl4*-deficient and ANGPTL4 antibody-treated animals on high-fat diets^7,8,22^. In an effort to determine if this finding has clinical relevance in humans, we evaluated the general health effects of homozygosity for the p.E40K variant by reviewing complete electronic health records available for 17 living individuals homozygous for p.E40K, including diagnosis and procedure codes, imaging and laboratory data, medication data and clinic notes covering a median of 6 years of clinical care (range 1–20 years). Five of these individuals had incidental abdominal computed tomography imaging performed; four of the radiologist reports commented specifically on normal abdominal lymph nodes, and the fifth reported normal findings with no comment on abdominal lymph nodes. A full summary of clinical findings is presented in Supplementary Data 1. These findings suggest that abdominal lymphadenopathy and related conditions are not common findings in humans homozygous for the K40 allele that abolishes ANGPTL4 ability to inhibit LPL.

To more comprehensively examine the clinical consequences of the p.E40K variant, we performed phenome-wide studies of association of p.E40K in 86,319 total individuals from the DiscovEHR discovery and replication cohorts. Using a Bonferroni significance threshold of 2.90 × 10^−5^ for associations with 1465 disease diagnoses and 261 clinical measurements from EHRs (Supplementary Data 2 and 3), we identified statistically significant associations with hyperglyceridemia, triglyceride, HDL-C, and non-HDL cholesterol levels, as well as leukocyte counts. These findings suggest that the clinical effects of the p.E40K variant may be specific to lipid levels and glucose homeostasis.

### *ANGPTL4* p.E40K and pLOFs abolish ANGPTL4 function

**Fig. 2:**
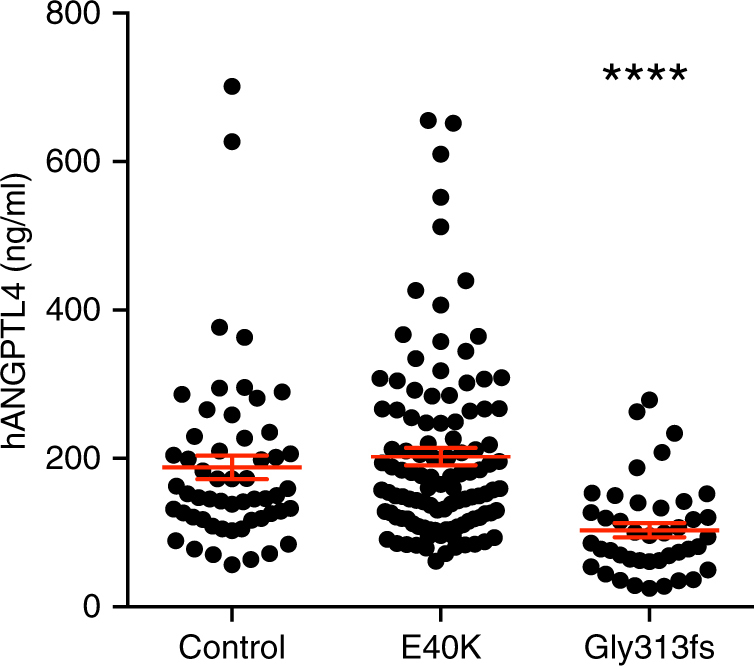
Plasma ANGPTL4 levels are reduced in p.G313fs carriers. ANGPTL4 plasma levels were measured in fasted serum from 86 heterozygous p.E40K, 42 heterozygous p.G313fs variant carriers, and 55 controls matched for age, sex, and body mass index. Statistics performed by unpaired *t*-test with Welch’s correction, comparing each variant carriers group to controls, ****p < 0.0001

To understand whether the p.E40K variant and the most abundant pLoF, p.G313fs, were associated with changes in circulating levels of ANGPTL4 protein in humans, we measured ANGPTL4 protein levels in available fasting serum samples from 86 p.E40K variant carriers, 42 p.G313fs variant carriers, and 55 non-carriers. Consistent with previous data^23^, the concentrations of ANGPTL4 protein in p.E40K variant carriers (203 ± 12 ng/ml) and in matched controls (188 ± 16 ng/ml) were not statistically different (Fig. 2). ANGPTL4 protein levels were reduced by 45% in heterozygous p.G313fs variant carriers (103.4 ± 9.5 ng/ml) compared to non-carriers. These findings are consistent with the expectation that heterozygous LoF genotype corresponds to complete loss of expression of one *ANGPTL4* allele.

**Fig. 3:**
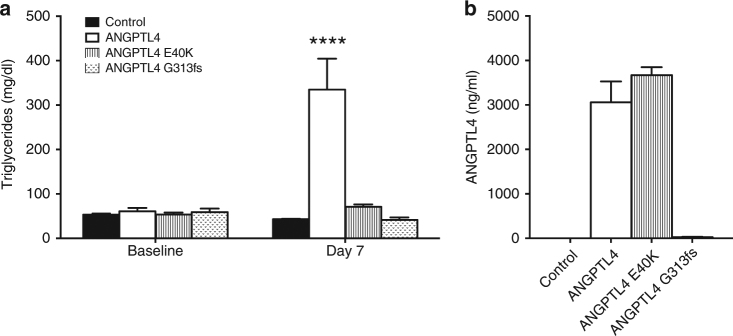
K40 and G313fs abolish ANGPTL4 effect on triglycerides. **a** Plasma triglycerides levels (4 h fasted) in C57Bl/6 mice before (Baseline) and 7 days after (Day 7) hydrodynamic delivery via tail vein injection of cDNA encoding human E40, K40, and Gly313fs ANGPTL4 variants. Control animals were injected with empty vector. **b** ANGPTL4 plasma levels were measured in the animals described in (**a**). All groups had five animals. Values are mean ± SEM. Statistics performed by two-way ANOVA with Bonferroni correction, ****p < 0.0001 vs control

To further understand the functional consequences of the p.E40K variant and the p.G313fs variant, we performed overexpression studies using hydrodynamic delivery of human *ANGPTL4* constructs to livers of chow-fed C57Bl/6 mice. These experiments revealed similarly increased plasma levels of E40 and K40 ANGPTL4. However, only E40 ANGPTL4 elicited an increase in plasma triglyceride levels, consistent with the observation that the p.E40K mutation prevents oligomerization of ANGPTL4, a step required for LPL inhibition^15^. Overexpression of ANGPTL4 with the Gly313fs variant did not increase plasma ANGPTL4 or triglyceride levels, suggesting this frame shift variant precludes ANGPTL4 presence in the circulation (Fig. 3). Collectively, these results suggest that p.E40K and p.G313fs both result in loss of ANGPTL4 expression and/or function.

### *Angptl4*-deficient mice have improved glucose homeostasis

**Fig. 4:**
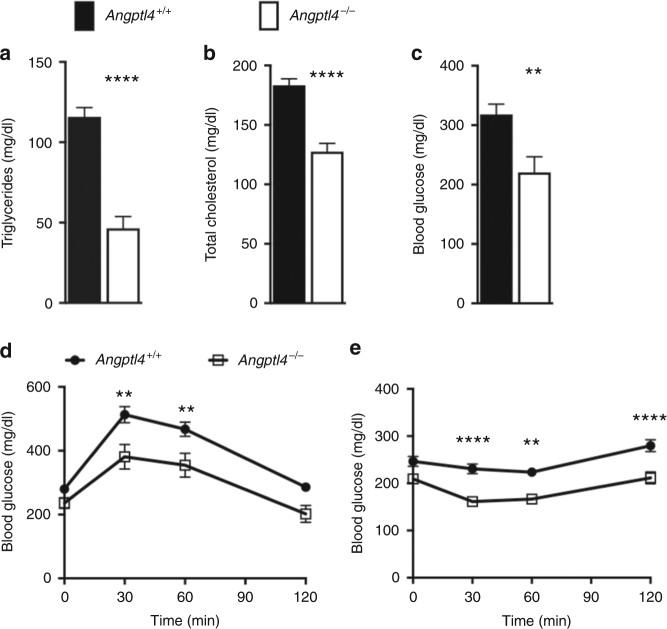
*Angptl4*^−/−^ mice have improved glucose homeostasis. **a** Serum triglycerides, (**b**) total cholesterol, and (**c**) blood glucose levels in *Angptl4*^−/−^ and littermate control mice on a high-fat diet for 9 weeks. **d** Oral glucose tolerance test and (**e**) insulin tolerance test in the animals described in (**a**–**c**). All groups had 9–11 animals. Values are mean ± SEM. Statistical analysis by Welch’s t-test (**a**) and 2-way ANOVA with Sidak’s post-test (**d**, **e**), **p < 0.001, ****p < 0.0001. The study was conducted in three different cohorts of mice, with qualitatively similar results in each replicate

To investigate the effects of genetic loss of ANGPTL4 function on glucose homeostasis in mice, we created *Angptl4*-deficient (*Angptl4*^−/−^) mice. Deletion of *Angptl4* led to significant reduction in circulating triglycerides as reported previously^4^ (Supplementary Fig. 1a and b). When placed on high-fat diet, *Angptl4*^−/−^ mice not only had reduced circulating triglycerides and cholesterol levels (Fig. 4a, b), but also had 31% lower non-fasted blood glucose (*Angptl4*^+/+^: 318 ± 17 mg/dl, *n* = 11; *Angptl4*^−/−^: 221 ± 26 mg/dl, *n* = 9) and improved glucose tolerance and insulin sensitivity (Fig. 4c–e). On chow diets, glucose levels and glucose tolerance were not significantly different between *Angptl4*^−/−^ and *Angptl4*^+/+^ littermates (Supplementary Fig. 1c-e).

**Fig. 5:**
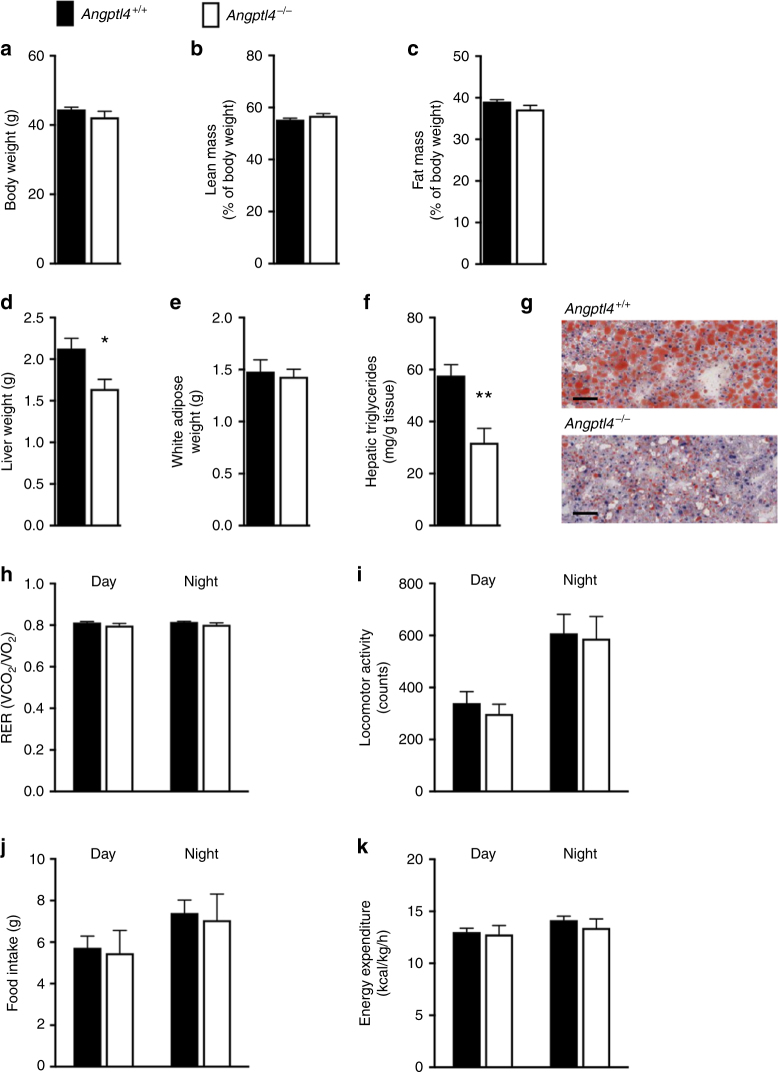
*Angptl4*^−/−^ mice have reduced liver fat. **a** Body weight, (**b**) lean and (**c**) fat mass in *Angptl4*^−/−^ and littermate control mice on a high-fat diet. **d** Liver and (**e**) epididymal white fat weights were evaluated in mice described in (**a–c**) at the time of killing. **f** Hepatic triglyceride levels and (**g**) neutral lipid staining in the livers described in (**d**), scale bar = 200 µm. **h** Respiratory exchange ratio (RER), (**i**) locomotor activity, (**j**) food intake, and (**k**) energy expenditure were measured during dark and light cycles in *Angptl4*^−/−^ and littermate control mice on a high-fat diet. All values are mean ± SEM. Statistical analysis was conducted by Welch’s t-test; ^*^p < 0.05; ^**^p < 0.01. The study was conducted in two different cohorts of mice, with qualitatively similar results in each replicate

The improvement in glucose homeostasis in high-fat fed *Angptl4*^−/−^ mice was not associated with changes in body weight or body composition when compared to their wildtype littermates (Fig. 5a–c, Supplementary Fig. 1f-h). Absolute liver weights and hepatic triglyceride accumulation were reduced in *Angptl4*^−/−^ mice after high-fat feeding (Fig. 5d, f), demonstrating that *Angptl4* deletion protects from fatty liver development. These findings were also supported by reduction of neutral lipids accumulation in the livers of *Angptl4*^−/−^ mice based on Oil Red O staining (Fig. 5g). Meanwhile, the epididymal fat pad weights showed no difference between the genotypes (Fig. 5e), suggesting normal peripheral fat storage.

To investigate the metabolic consequences of *Angptl4* deletion, high-fat fed *Angptl4*^−/−^ mice and their wildtype littermates were placed into metabolic cages. The analysis revealed no differences in O_2_ consumption, CO_2_ production (Supplementary Fig. 2a and b) respiratory quotient, activity, food intake, or energy expenditure between the genotypes (Fig. 5h–k). At the end of the study (9 weeks on high-fat diet), the *Angptl4*^−/−^ mice were in good health, as judged by comparable weight gain, activity, energy expenditure, and food intake to control mice (Fig. 2). Furthermore, no signs of intestinal abnormalities or lethality were observed. Collectively, these findings show that genetic deficiency of *Angptl4* improves glucose homeostasis and insulin sensitivity without associated changes in body weight, composition or metabolism.

## Discussion

In summary, by sequencing the exons of *ANGPTL4* in 58,124 participants in the DiscovEHR study population, we identified 2235 heterozygotes and 26 homozygotes for the p.E40K variant that has been shown to have decreased ability to inhibit LPL function^15^. We identified 21 additional variants that were predicted to inactivate one allele of *ANGPTL4*. Linking genetic variants to EHR-derived phenotypes, we found that p.E40K and other pLoF variants in *ANGPTL4* were associated with reduced risk of type 2 diabetes, with p.E40K reaching genome-wide significance in analysis of 95,711 type 2 diabetes cases and 534,926 controls, and pLoFs reaching nominal significance in analyses of exome data from 32,015 type 2 diabetes cases and 84,006 controls. These findings confirm and extend findings from a recent report of reduced type 2 diabetes risk associated with p.E40K^14^. Further, the p.E40K variant was nominally associated with lower fasting glucose in non-diabetic humans and improved insulin sensitivity in oral glucose tolerance tests. A gain-of-function genetic variant in *LPL* has been reported to be associated with lower fasting insulin and type 2 diabetes risk^24^, indicating that genetic modulation of lipoprotein lipase activity affects insulin sensitivity and diabetes risk in humans. Whether the association of genetic variants that inactivate ANGPTL4 with reduced type 2 diabetes risk is mediated exclusively via modulation of lipoprotein lipase activity or involves other mechanisms remains to be clarified.

Furthermore, we found that deletion of *Angplt4* in mice fed a high-fat diet improved insulin sensitivity and glucose homeostasis in fasting and post-prandial states. Although the mechanism linking loss of ANGPTL4 function to improved insulin sensitivity and glucose homeostasis is not completely understood, our animal studies revealed that improved glucose homeostasis with *Angptl4* deletion occurs without changes in body weight, body fat content or energy metabolism. We also observed a reduction in triglyceride accumulation in the livers of the *Angptl4*^−*/*−^ mice when placed on a high-fat diet. These findings are consistent with recent findings in adipose-specific *Angptl4* knockout mice^25^. The authors reported increased plasma fatty acids uptake by adipose tissue, along with increased triglyceride lipolysis and oxidation leading to reduction in ectopic lipids accumulation in liver and skeletal muscle, and concomitant improvement in glucose tolerance and insulin sensitivity.

Our data, in combination with findings of favorable lipid profiles and reduced risk of coronary artery disease^5,6,8^, provide human genetics support for ANGPTL4 inhibition as a therapeutic strategy for prevention and possibly treatment of metabolic disease. The observation of an association between protein-truncating genetic variants in *SLC30A8* and reduced risk of type 2 diabetes has illuminated a new therapeutic target opportunity for type 2 diabetes^26^. A genetic variant in *GLP1R* that mimics the glycemic effects of the Food and Drug Administration (FDA)-approved antidiabetic glucagon-like peptide-1 receptor agonists^27^ has also been shown to reduce coronary heart disease risk^28^. We describe *ANGPTL4* as a gene in which loss-of-function variants confer protection from both coronary atherosclerosis and type 2 diabetes. Concerns remain about therapeutic modulation of ANGPTL4 related to observations of abdominal lymphadenopathy in *Angptl4*-deficient and ANGPTL4 antibody-treated animals^7,8,22^. In a small number of human patients homozygous for K40, abdominal imaging did not demonstrate lymphadenopathy, raising the question of whether this concern is relevant in humans. It is not yet clear whether a larger sampling of individuals deficient in ANGPTL4 activity would reveal abdominal lymphadenopathy, or whether therapeutic antagonism of ANGPTL4 late in life will have similar effects in humans.

Our study has important limitations. We focused on type 2 diabetes risk in individuals with a known reduced-function allele (p.E40K) and among carriers of splice-disrupting, frame shifting, premature stop, and start or stop loss variants. Other coding or regulatory non-coding variants might yield additional insight into the effects, in humans, of ANGPTL4 antagonism. Our study was performed in individuals of primarily European ancestry; it is not known whether our observations will generalize to individuals from other ancestries. Finally, the metabolic and diabetes associations were primarily driven by phenotypic observations in heterozygous carriers of loss-of-function variants. Whether such observations will accurately reflect the phenotypic effects of more complete therapeutic blockade is not clear.

In conclusion, we found that loss-of-function variants in *ANGPTL4* were associated with increased insulin sensitivity and reduced risk of type 2 diabetes, mirroring the beneficial effects on glucose homeostasis of deletion of *Angptl4* in mice. ANGPTL4 may be a promising target for therapeutic inhibition for reduction of metabolic disease risk in humans.

## Methods

### Study oversight of human genetics studies

We conducted human genetics studies using DNA samples and data from 13 study cohorts. The DiscovEHR study was performed by the Regeneron Genetics Center and the Geisinger Health System, and the Regeneron Genetics Center funded study sample collection, sequence data generation, and clinical and sequence data analysis for DiscovEHR. All human subject research was approved by the relevant institutional review boards, and all participants gave informed written consent.

### Sequencing of *ANGPTL4* at the Regeneron Genetics Center®

Sequence data for *ANGPTL4* were extracted from exome sequences generated at the Regeneron Genetics Center® with the use of protocols as described^8,29^. Briefly, genomic DNA was sheared to an average fragment length of 150 base pairs and prepared for exome capture with NimbleGen probes (SeqCap VCRome) or xGen Exome Research Panel v1.0 (Integrated DNA Technologies). Captured DNA was amplified, quantified, and subsequently sequenced on an Illumina v4 HiSeq 2500 instrument (to approximately 80× mean haploid read depth of targeted bases). Sequence reads were aligned to the human reference build GRCh38, and single-nucleotide variants (SNVs) and insertion/deletion (indel) sequence variants were identified using the Genome Analysis Toolkit^30^, and annotated using SnpEff^31^.

Variants in *ANGPTL4* were identified via positional intersection with Ensembl transcript ENST00000301455 (RefSeq mRNA sequence NM_139314). The following variants were defined as predicted loss-of-function (pLoF) variants: SNVs leading to loss of a start codon, or loss of a stop codon, or to a premature stop codon; open reading frame shifting indels leading to the formation of a premature stop codon; and SNVs or indels disrupting canonical splice acceptor or donor dinucleotides. Carriers of the missense mutation p.E40K (rs116843064), which has been previously characterized functionally as a reduced-function variant^15^, were also identified using the SnpEff annotations. Sanger sequencing of pLoF variant regions was performed as described for select variants, focusing on indel variants.

### Association of p.E40K with type 2 diabetes and glucose

Thirteen study populations were used to evaluate associations of p.E40K with type 2 diabetes. Six of these study populations (DiscovEHR study, DiscovEHR replication cohort, UPenn study, the T2D-GENES, GoT2D, and DIAGRAM studies, the Malmo Diet and Cancer Study, and the HUNT study) were used to evaluate associations of rs116843064 with fasting glucose in non-diabetic individuals. A summary of studies, including definitions for type 2 diabetes case status, is provided in Supplementary Table 4.

The DiscovEHR human genetics study population for this analysis included: (1) 58,124 consented enrollees of European ancestry from the ongoing MyCode Community Health Initiative in the Geisinger Health System (“DiscovEHR study”) that were used for primary association discovery; and (2) an additional 28,915 exome-sequenced DiscovEHR study participants of European ancestry (“DiscovEHR replication cohort”). Participants were recruited from outpatient primary care and specialty clinics, the cardiac catheterization laboratory, and from patient populations referred for bariatric and abdominal vascular surgery between 2007 and 2016. Clinical laboratory measurements, International Classification of Diseases, Ninth Revision (ICD-9) disease diagnosis codes, medications, and procedural codes were extracted from the EHR recording a median of 15 years of clinical care.

Type 2 diabetes was defined using a modified version of the Electronic Medical Records and Genomics (eMERGE) Network type 2 diabetes electronic phenotyping algorithm^32^. In brief, patients were considered to have type 2 diabetes if they had at least two out of (1) a diagnosis of type 2 diabetes in the electronic health record, (2i) antidiabetic medication use, or (3) fasting glucose greater than 126 mg/dl or hemoglobin A1c greater than 6.5%. Patients who met criteria (2) and (3) and who had a type 1 diabetes diagnosis only were excluded. Type 2 diabetes controls were patients who had met none of the three inclusion criteria.

Median values for serially measured laboratory and anthropometric traits, including BMI and fasting glucose were calculated for all individuals with two or more measurements in the EHR following removal of likely spurious values that were greater than three standard deviations from the intra-individual median value. BMI values obtained during pregnancy were excluded.

We performed association analysis using the Mixed Model Analysis for Pedigrees (MMAP) (https://mmap.github.io/) software, which accounts for the relatedness of study subjects by conditioning the genotype–phenotype correlations on the phenotypic correlations among relative pairs. For both the discovery and replication cohorts, genetic relatedness matrices (GRMs) and principal components (PCs) were calculated using autosomal SNVs with MAF ≥5% and in approximate linkage equilibrium (LD pruning performed in PLINK using the --indep-pairwise command with window size of 50 variants, step size of 5 variants, and *r*^2^ threshold of 0.5), excluding variants in the major histocompatibility complex and other high complexity regions of the genome.

We used linear mixed models (LMMs) of association to test for associations between log-transformed median glucose (discovery cohort) or untransformed median glucose (replication cohort) values and genotype for aggregated *ANGPTL4* pLoFs and p.E40K. We generated residuals adjusted for age, age^2^, sex, and the first four PCs of ancestry. Trait residuals were tested for association with genotype under an additive genetic model, and a study-specific GRM, which captures population structure from ancestry and relatedness, was included in each model as a random-effects covariate. We tested for associations between genotype and type 2 diabetes disease status in each cohort using linear mixed models adjusted for age, age^2^, sex, the first four PCs of ancestry, and the study-specific GRM; throughout the manuscript we present *p* values from LMM analyses. To obtain interpretable effect estimates for binary traits, we additionally performed tests of association (adjusting for age, age^2^, sex, and the first four PCs of ancestry) using Firth’s penalized likelihood logistic regression^33^ to estimate odds ratios, and estimated Wald 95% confidence intervals using standard error estimates back calculated from *p* values from the mixed linear models of association.

In the T2D-Genes/GoT2D/DIAGRAM studies, a combined analysis was performed of rs116843064 genotypes extracted from exome array and whole exome sequencing data for 72,803 participants in the T2D-Genes/GoT2D/DIAGRAM studies^34^. The analysis of type 2 diabetes was performed using logistic regression adjusted for age, sex, and intra-ethnic PCs followed by meta-analysis, with no further adjustment. Fasting glucose was analyzed in non-diabetics using linear regression adjusted for the same covariates.

The Michigan Genomics Initiative (MGI) is an institutional repository of DNA and genetic data for broad long-term use at the University of Michigan. All pre-operative patients 18 years of age or older are eligible for participation in the Medical School Central Biorepository, which involves collecting blood and completing a short questionnaire regarding pre-operative pain and basic lifestyle questions, and links to electronic health record data. Exome array data were available for 13,678 individuals of European ancestry who met case or control criteria for type 2 diabetes. Firth’s penalized likelihood logistic regression adjusted for sex and two PCs of ancestry was used to test for an association between rs116843064 genotype (coded using an additive model) and type 2 diabetes.

Exome array data were available for 27,914 individuals participating in the prospective Malmo and Diet and Cancer cohort study^35^. Logistic regression adjusted for age, age^2^, and sex was used to test for an association between rs116843064 genotype (coded using an additive model) and type 2 diabetes, defined as previously described^36^. Linear regression adjusted for the same covariates was used to test for an association between rs116843064 and log_10_ transformed fasting glucose in 4848 individuals.

The UK Biobank is a prospective study of >500,000 people living in the United Kingdom aged 40–69 years and living <25 miles from a study center. Analysis was restricted to the subset of participants of white British descent, derived by UK Biobank. Related individuals, individuals whose genetic sex did not match self-reported sex, and extreme outliers were excluded, leaving 112,338 participants for analysis. A combined analysis was performed of rs116843064 genotype data extracted from genotype data produced for 112,338 UK Biobank^37^ study participants using the UK Biobank array and UK BiLEVE arrays. Logistic regression adjusted for age, sex, and 10 PCs of ancestry was used to test for an association between rs116843064 and type 2 diabetes.

The Nord-Trøndelag Health Study (The HUNT Study) is a population-based health survey of more than 120,000 individuals conducted in the county of Nord-Trøndelag in Norway from 1984 to 2016^38^. The database contains clinical examination results and is additional data from regional-level cross referencing with registries. Genotype data for rs116843064 were available for 61,598 individuals of Norwegian European ethnicity. Logistic regression adjusted for age, age^2^, sex, and PCs 1–4 were used for assessing the association between rs116843064 and type 2 diabetes, applying an additive genetic model. Fasting glucose (>8 h since last meal) were available for 2714 individuals without type 2 diabetes. Linear regression adjusted for the aforementioned covariates was used to test for the association between rs116843064 and log_10_ transformed fasting glucose measured in mmol/l.

The EPIC-InterAct case-cohort study^39^ is nested within the European Prospective Investigation into Cancer and Nutrition (EPIC) cohorts^40^. EPIC-InterAct includes 12,403 incident T2D cases and a subcohort of 16,154 individuals that includes 778 randomly selected incident T2D cases. rs116843064 was either directly genotyped on the Illumina HumanCoreExome chip, or imputed from genotyping on the Illumina Quad660 genotyping chip (info = 0.87). Individuals in the EPIC-Norfolk study were removed from analyses in EPIC-InterAct to avoid double-counting. Association analysis with type 2 diabetes was performed using logistic regression adjusted for age, age^2^, sex, and PCs 1–4.

EPIC-Norfolk is a prospective cohort study of 25,639 individuals aged between 40 and 79 years and living in the Norfolk county in East Anglia (United Kingdom) at recruitment^41^. EPIC-Norfolk is a constituent cohort of the European Prospective Investigation of Cancer. Participants were genotyped on the Affymentrix UKBiobank chip, on which the p.E40K variant was directly genotyped. Association analysis with type 2 diabetes was performed using logistic regression adjusted for age, age^2^, sex, and PCs 1–4.

A total of 107,834 individuals from the Copenhagen City Heart Study, Copenhagen General Population Study, and Copenhagen Ischemic Heart Disease Study were included in the analysis^42,43^. Participants were directly genotyped for rs116843064 with the use of the ABI PRISM 7900HT Sequence Detection System (Applied Biosystems) and TaqMan-based assays or with the use of an allele-specific PCR system (KASPer, LGC Genomics). Association analysis with type 2 diabetes was performed using logistic regression adjusted for age, age^2^, and sex.

The deCODE study population for these analyses comprises 79,117 Icelandic participants genotyped directly or with imputed (using long-range phased haplotypes and, for a subset of cases, on the basis of information from genotyped close relatives) genotypes for rs116843064. The rs116843064 variant was imputed accurately in the deCODE population (imputation info = 0.99). The type 2 diabetes case definition is described in Supplementary Table 4. Briefly, cases were enrolled on the basis of four different, partially overlapping criteria: (1) Clinician confirmed T2D cases; (2) T2D oral medication three times or more, or two times and current; (3) two or more measures of hemoglobin A1c (HbA1c) >6.5%; and (4) one measure of HbA1c >6.5% and oral medication and either self-reported T2D diagnoses or hospital discharge diagnoses of T2D (PMID: 24464100). HbA1c levels were extracted from computerized laboratory data. Participants with type 1 diabetes were excluded. A control group was comprised of individuals recruited for genetic research projects at deCODE without T2D. A generalized form of logistic regression was used to test for association with sequence variants^44^. Sex, county of birth, current age or age at death (first- and second-order terms), blood sample availability, and an indicator function for the overlap of the individual’s lifespan with the timespan of phenotype collection were included in the association model as nuisance variables. The T2D association results were genomic controlled using LD score regression (lambda = 1.53)^45^.

Fasting glucose levels were measured in 39,700 non-diabetic Icelanders (33,085 with available array genotype data). The measurements were performed for clinical indications and were obtained from three of the largest laboratories in Iceland: Landspitali University Hospital (LUH) in Reykjavik, Iceland, The Icelandic Medical Center (Laeknasetrid) laboratory in Mjodd, Reykjavik, Iceland, and The Regional Hospital in North Iceland (FSA), Akureyri, Iceland. Fasting glucose levels were quantile–quantile standardized and age-adjusted for each sex separately. A generalized linear regression model (mixed effect model) was used to test for associations between sequence variants and quantitative traits, assuming an additive genetic model^46^. The fasting glucose association results were genomic controlled using LD-score regression (lambda = 1.33)^45^.

Participants in the Penn Medicine Biobank were recruited from phlebotomy labs, preadmission testing, and cardiac catheterization labs at the University of Pennsylvania Health System and consented for biospecimen storage, access to EHR data, and permission to recontact. A total of 8123 participants of European ancestry were included in this analysis. Type 2 diabetes case status was defined as in Supplementary Table 4. rs116843064 genotypes were extracted from exome sequence data generated at the Regeneron Genetics Center® according to the protocols described above. Genetic association analyses for glucose and type 2 diabetes disease status were performed as described for the DiscovEHR discovery and replication cohorts (described above).

A total of 6533 individuals from the Duke CATHeritization GENetics (CATHGEN) bio-repository were included in this analysis. CATHGEN includes clinical data and biological samples from individuals undergoing cardiac catheterization between 2001 and 2010^47^. Type 2 diabetes disease case status was defined as in Supplementary Table 4. rs116843064 genotype LoFs were extracted from exome sequence data generated at the Regeneron Genetics Center according to protocols described above, with the following modification: samples were processed using the Kapa HyperPlus kit modified for the Regeneron Genetics Center’s custom automation and captured with the xGen Exome Research Panel v1.0 from Integrated DNA Technologies (IDT). Genetic association analyses for type 2 diabetes disease status were performed as described for the DiscovEHR discovery and replication cohorts (described above).

Since phenotype transformations differed across contributing studies, single-study summary statistics for the p.E40K association with glucose were combined for meta-analysis using sample size-weighted *p* value-based meta-analysis in METAL^48^, taking into account the direction of effect for each study. Summary statistics for the p.E40K association with type 2 diabetes were combined using inverse variance weighted meta-analysis in METAL.

### Association of pLoFs in *ANGPTL4* with type 2 diabetes

A summary of studies, including definitions for type 2 diabetes case status, for the association between *ANGPTL4* pLoFs and type 2 diabetes is provided in Supplementary Table 7.

For the DiscovEHR study and DiscovEHR replication cohort, *ANGPTL4* pLoFs were extracted from exome sequence data generated at the Regeneron Genetics Center® according to the protocols described above for the DiscovEHR study and DiscovEHR replication cohort. *ANGPTL4* pLoF carrier status and type 2 diabetes case status were tabulated.

For UPenn Medicine Biobank, *ANGPTL4* pLoFs were extracted from exome sequence data generated at the Regeneron Genetics Center® according to the protocols as described above for the UPenn Medicine Biobank cohort. *ANGPTL4* pLoF carrier status and type 2 diabetes case status were tabulated.

For Duke CATHGEN, *ANGPTL4* pLoFs were extracted from exome sequence data generated at the Regeneron Genetics Center according to the protocols described above for Duke CATHGEN cohort. *ANGPTL4* pLoF carrier status and type 2 diabetes case status were tabulated.

ForTAICHI, a total of 9058 samples from the TAIwan metaboCHIp (TAICHI) consortium^49–51^, which aims to identify genetic determinants of atherosclerosis- and metabolic-related traits in Taiwanese Chinese, were included in this analysis. Academic centers participating include Taichung Veteran’s General Hospital, Tri-Service General Hospital and the National Taiwan University Hospital, and the National Health Research Institute in Taiwan for subject ascertainment and phenotyping. Type 2 diabetes case status was defined according to physician diagnosis criteria and adjudicated electronic health record information. *ANGPTL4* pLoFs were extracted from exome sequence data generated at the Regeneron Genetics Center® according to the protocols described above, and *ANGPTL4* pLoF carrier status and type 2 diabetes case status were tabulated.

In T2D-Genes/GoT2D/DIAGRAM studies, *ANGPTL4* pLoFs were extracted from exome sequence data generated on a subset of 16,839 participants in the T2D-Genes/GoT2D/DIAGRAM studies described above and by Fuchsberger et al.^34^. *ANGPTL4* pLoF carrier status and type 2 diabetes case status were tabulated.

The Dallas Heart Study is a probability-based population cohort study of Dallas County residents aged 30 to 65 years^52^. The Dallas Heart Study population used for this analysis was comprised of 1355 European Americans and 2385 African Americans. *ANGPTL4* pLoFs were extracted from exome sequence data generated at the Regeneron Genetics Center® according to the protocols described above, and *ANGPTL4* pLoF carrier status and type 2 diabetes case status were tabulated.

Since the very small counts of carriers of pLoF variants prohibited meta-analysis of summary statistics, we assessed the association of *ANGPTL4* pLOF variants with type 2 diabetes by performing a two-sided exact conditional test of marginal counts of carriers by type 2 diabetes disease status (implemented in the mantelhaen. test function in the base R stats package; R Core Team (2017). R: A language and environment for statistical computing. R Foundation for Statistical Computing, Vienna, Austria. URL https://www.R-project.org/).

### Association of p.E40K with oral glucose tolerance

We performed tests of association of p.E40K, encoded using an additive genetic model, with measures of tolerance to a 75 g oral glucose challenge in up to 8081 non-diabetic participants in the NIDDM Botnia^20^ and Prevalence, Prediction, and Prevention of Diabetes-Botnia^21^ studies. All the traits were rank inverse transformed and converted to *Z* score units. Association analyses were performed using linear mixed models of association adjusted for age, sex and BMI. A genetic relatedness matrix was included in each model as a random-effects covariate.

### Phenome-wide studies of association of p.E40K

We performed a phenome-wide study of associations of p.E40K with 316 quantitative EHR-derived anthropometric, vital sign, laboratory, electrocardiographic, echocardiographic, and bone densitometry measurements, and also with 1585 EHR-derived clinical diagnoses. Median laboratory values for individuals with serial outpatient measures were calculated following removal of likely spurious values that were >3 standard deviations from the intra-individual median value; maximum and minimum values were also calculated. We then calculated trait residuals for all laboratory traits after adjustment for age, age^2^, sex, and the first four principal components, and applied appropriate transformations prior to association analysis. ICD-10 diagnosis codes were mapped to hierarchical clinical disease case groups and corresponding control groups using a modified version of the groupings proposed by Denny et al.^53,54^. ICD-10-based diagnoses required a problem list entry of the diagnosis code or an encounter diagnosis code entered for two separate clinical encounters on separate calendar days.

Analyses of association with transformed quantitative clinical measurement residuals were performed using linear regression, and analyses of association with clinical diagnoses were performed using Firth’s penalized likelihood logistic regression adjusted for age, age^2^, sex, and the first four principal components. Alleles were coded using the additive (0 for reference allele homozygotes, 1 for heterozygotes, and 2 for alternative allele homozygotes) model.

### Animal studies

*Angptl4*^−/−^ mice (99.9% C578Bl/6NTac background) were generated using Regeneron’s VelociGene technology^55^. Male mice, single housed at 6–9 weeks of age, were maintained on a 12 h light/dark cycle and and fed ad libitum with chow (LabDiet, 5001) or high-fat diet (Research Diets, D12451; 45% fat by calories). Glucose tolerance and glucose levels were measured as previously described^56^. Circulating triglycerides, total cholesterol, alanine aminotransferase, and aspartate aminotransferase levels were determined in serum using an ADVIA^®^ 1800 blood chemistry analyzer (Siemens). Liver triglyceride level was evaluated as previously described^57^. Body composition was measured using PIXIMus dual energy X-ray absorptiometry (DEXA) (GE Medical Systems). Metabolic cage data were generated using the Oxymax Lab Animal Monitoring System CLAMS (Columbus Instruments) as described^58^. The overexpression studies using hydrodynamic DNA delivery were conducted as previously described^56^.

All animal procedures were conducted in compliance with protocols approved by the Regeneron Pharmaceuticals Institutional Animal Care and Use Committee. For the mouse, data are expressed as mean +/− standard error of the mean. Mean values were compared using unpaired *t*-tests or two-way analysis of variance (ANOVA) as implemented in the Graphpad Prism 6.0 software (Graphpad Software, Inc.).

### Evaluation of ANGPTL4 concentration in human plasma

Human ANGPTL4 plasma levels were measured by hANGPTL4 ELISA (DY3485, R&D Systems, MN).

### Data availability

The data supporting the findings of this study are available within the article and its Supplementary Data files. Additional information for reproducing the results described in the article is available upon reasonable request and subject to a data use agreement. Additional information on the DiscovEHR study is available at http://www.discovehrshare.com.

## Electronic supplementary material

Supplementary Information

Description of Additional Supplementary Files

Supplementary Data 1

Supplementary Data 2

Supplementary Data 3

## References

[1] Yoshida K, Shimizugawa T, Ono M, Furukawa H (2002). Angiopoietin-like protein 4 is a potent hyperlipidemia-inducing factor in mice and inhibitor of lipoprotein lipase. J. Lipid Res..

[2] Yau MH (2009). A highly conserved motif within the NH2-terminal coiled-coil domain of angiopoietin-like protein 4 confers its inhibitory effects on lipoprotein lipase by disrupting the enzyme dimerization. J. Biol. Chem..

[3] Sukonina V, Lookene A, Olivecrona T, Olivecrona G (2006). Angiopoietin-like protein 4 converts lipoprotein lipase to inactive monomers and modulates lipase activity in adipose tissue. Proc. Natl. Acad. Sci. USA.

[4] Koster A (2005). Transgenic angiopoietin-like (angptl)4 overexpression and targeted disruption of angptl4 and angptl3: regulation of triglyceride metabolism. Endocrinology.

[5] Romeo S (2007). Population-based resequencing of ANGPTL4 uncovers variations that reduce triglycerides and increase HDL. Nat. Genet..

[6] Romeo S (2009). Rare loss-of-function mutations in ANGPTL family members contribute to plasma triglyceride levels in humans. J. Clin. Invest.

[7] Desai U (2007). Lipid-lowering effects of anti-angiopoietin-like 4 antibody recapitulate the lipid phenotype found in angiopoietin-like 4 knockout mice. Proc. Natl. Acad. Sci. USA.

[8] Dewey FE (2016). Inactivating variants in ANGPTL4 and risk of coronary artery disease. N. Engl. J. Med..

[9] Myocardial Infarction Genetics and CARDIoGRAM Exome Consortia Investigators. (2016). Coding variation in ANGPTL4, LPL, and SVEP1 and the risk of coronary disease. N. Engl. J. Med..

[10] Wang Y (2015). Hepatic ANGPTL3 regulates adipose tissue energy homeostasis. Proc. Natl. Acad. Sci. USA.

[11] Zhang R (2016). The ANGPTL3-4-8 model, a molecular mechanism for triglyceride trafficking. Open Biol..

[12] Kersten S (2014). Physiological regulation of lipoprotein lipase. Biochim. Biophys. Acta.

[13] Dijk W, Kersten S (2016). Regulation of lipid metabolism by angiopoietin-like proteins. Curr. Opin. Lipidol..

[14] Liu DJ (2017). Exome-wide association study of plasma lipids in 300,000 individuals. Nat. Genet.

[15] Yin W (2009). Genetic variation in ANGPTL4 provides insights into protein processing and function. J. Biol. Chem..

[16] Okamoto H (2017). Angptl4 does not control hyperglucagonemia or alpha-cell hyperplasia following glucagon receptor inhibition. Proc. Natl. Acad. Sci. USA.

[17] Xu A (2005). Angiopoietin-like protein 4 decreases blood glucose and improves glucose tolerance but induces hyperlipidemia and hepatic steatosis in mice. Proc. Natl. Acad. Sci. USA.

[18] Wang Y (2016). Angiopoietin-like protein 4 improves glucose tolerance and insulin resistance but induces liver steatosis in high-fat-diet mice. Mol. Med. Rep..

[19] Mandard S (2006). The fasting-induced adipose factor/angiopoietin-like protein 4 is physically associated with lipoproteins and governs plasma lipid levels and adiposity. J. Biol. Chem..

[20] Groop L (1996). Metabolic consequences of a family history of NIDDM (the Botnia study): evidence for sex-specific parental effects. Diabetes.

[21] Isomaa B (2010). A family history of diabetes is associated with reduced physical fitness in the Prevalence, Prediction and Prevention of Diabetes (PPP)-Botnia study. Diabetologia.

[22] Lichtenstein L (2010). Angptl4 protects against severe proinflammatory effects of saturated fat by inhibiting fatty acid uptake into mesenteric lymph node macrophages. Cell Metab..

[23] Smart-Halajko MC (2010). The relationship between plasma angiopoietin-like protein 4 levels, angiopoietin-like protein 4 genotype, and coronary heart disease risk. Arterioscler. Thromb. Vasc. Biol..

[24] Lotta LA (2017). Integrative genomic analysis implicates limited peripheral adipose storage capacity in the pathogenesis of human insulin resistance. Nat. Genet.

[25] Aryal B (2018). Absence of ANGPTL4 in adipose tissue improves glucose tolerance and attenuates atherogenesis. JCI Insight.

[26] Flannick J (2014). Loss-of-function mutations in SLC30A8 protect against type 2 diabetes. Nat. Genet..

[27] Lovshin JA, Drucker DJ (2009). Incretin-based therapies for type 2 diabetes mellitus. Nat. Rev. Endocrinol..

[28] Scott RA (2016). A genomic approach to therapeutic target validation identifies a glucose-lowering GLP1R variant protective for coronary heart disease. Sci. Transl. Med..

[29] Dewey FE (2016). Distribution and clinical impact of functional variants in 50,726 whole-exome sequences from the DiscovEHR study. Science.

[30] McKenna A (2010). The Genome Analysis Toolkit: a MapReduce framework for analyzing next-generation DNA sequencing data. Genome Res..

[31] Cingolani P (2012). A program for annotating and predicting the effects of single nucleotide polymorphisms, SnpEff: SNPs in the genome of Drosophila melanogaster strain w1118; iso-2; iso-3. Fly (Austin).

[32] Kho AN (2012). Use of diverse electronic medical record systems to identify genetic risk for type 2 diabetes within a genome-wide association study. J. Am. Med. Inform. Assoc..

[33] Heinze G, Schemper M (2002). A solution to the problem of separation in logistic regression. Stat. Med..

[34] Fuchsberger C (2016). The genetic architecture of type 2 diabetes. Nature.

[35] Berglund G, Elmstahl S, Janzon L, Larsson SA (1993). The Malmo Diet and Cancer Study. Design and feasibility. J. Intern. Med..

[36] Enhorning S, Hedblad B, Nilsson PM, Engstrom G, Melander O (2015). Copeptin is an independent predictor of diabetic heart disease and death. Am. Heart J..

[37] Allen NE, Sudlow C, Peakman T, Collins R, Biobank UK (2014). UK biobank data: come and get it. Sci. Transl. Med..

[38] Krokstad S (2013). Cohort Profile: the HUNT Study, Norway. Int J. Epidemiol..

[39] InterAct C (2011). Design and cohort description of the InterAct Project: an examination of the interaction of genetic and lifestyle factors on the incidence of type 2 diabetes in the EPIC Study. Diabetologia.

[40] Riboli E (2002). European Prospective Investigation into Cancer and Nutrition (EPIC): study populations and data collection. Public Health Nutr..

[41] Day N (1999). EPIC-Norfolk: study design and characteristics of the cohort. European Prospective Investigation of Cancer. Br. J. Cancer.

[42] Jorgensen AB (2013). Genetically elevated non-fasting triglycerides and calculated remnant cholesterol as causal risk factors for myocardial infarction. Eur. Heart J..

[43] Varbo A (2013). Remnant cholesterol as a causal risk factor for ischemic heart disease. J. Am. Coll. Cardiol..

[44] Steinthorsdottir V (2014). Identification of low-frequency and rare sequence variants associated with elevated or reduced risk of type 2 diabetes. Nat. Genet..

[45] Bulik-Sullivan BK (2015). LD Score regression distinguishes confounding from polygenicity in genome-wide association studies. Nat. Genet..

[46] Helgadottir A (2016). Variants with large effects on blood lipids and the role of cholesterol and triglycerides in coronary disease. Nat. Genet..

[47] Kraus WE (2015). A Guide for a Cardiovascular Genomics Biorepository: the CATHGEN Experience. J. Cardiovasc Transl. Res..

[48] Willer CJ, Li Y, Abecasis GR (2010). METAL: fast and efficient meta-analysis of genomewide association scans. Bioinformatics.

[49] Kuo JZ (2013). Trans-ethnic fine mapping identifies a novel independent locus at the 3’ end of CDKAL1 and novel variants of several susceptibility loci for type 2 diabetes in a Han Chinese population. Diabetologia.

[50] Sheu WH (2013). Genome-wide association study in a Chinese population with diabetic retinopathy. Hum. Mol. Genet..

[51] Chang YC (2012). Replication of genome-wide association signals of type 2 diabetes in Han Chinese in a prospective cohort. Clin. Endocrinol. (Oxf.).

[52] Victor RG (2004). The Dallas Heart Study: a population-based probability sample for the multidisciplinary study of ethnic differences in cardiovascular health. Am. J. Cardiol..

[53] Denny JC (2013). Systematic comparison of phenome-wide association study of electronic medical record data and genome-wide association study data. Nat. Biotechnol..

[54] Denny JC (2010). PheWAS: demonstrating the feasibility of a phenome-wide scan to discover gene-disease associations. Bioinformatics.

[55] Valenzuela DM (2003). High-throughput engineering of the mouse genome coupled with high-resolution expression analysis. Nat. Biotechnol..

[56] Gusarova V (2014). ANGPTL8/betatrophin does not control pancreatic beta cell expansion. Cell.

[57] Gusarova V (2015). ANGPTL3 blockade with a human monoclonal antibody reduces plasma lipids in dyslipidemic mice and monkeys. J. Lipid Res..

[58] Gusarova V (2017). ANGPTL8 blockade with a monoclonal antibody promotes triglyceride clearance, energy expenditure, and weight loss in mice. Endocrinology.

