# Supplementary material for: Genetic inactivation of ANGPTL4 improves glucose homeostasis and is associated with reduced risk of diabetes
Source: Nat Commun. 2018 Jun 13;9:2252. doi: 10.1038/s41467-018-04611-z (PMC5997992; doi:10.1038/s41467-018-04611-z)
Supplement: Supplementary file 1 — Supplementary Information [file 41467_2018_4611_MOESM1_ESM.pdf]

Supplementary Information

Gusarova V, et al. Genetic Inactivation of ANGPTL4 Improves Glucose Homeostasis and Reduces Risk of Type 2 Diabetes.

Supplementary Note 1

Additional Acknowledgements:

DiscovEHR: The authors would like to thank the MyCode® Community Health Initiative participants for their permission to utilize their health and genomics information in the DiscovEHR collaboration. The DiscovEHR study was funded in whole by Regeneron Pharmaceuticals.

TAICHI Study: The TAICHI study was supported by grants from the National Health Research Institutes, Taiwan (PH-099-PP-03, PH-100-PP-03, and PH-101-PP-03); the National Science Council, Taiwan (NSC 101-2314-B-075A-006-MY3, MOST 104-2314-B 075A-006-MY3, MOST 104-2314-B-075A-007, and MOST 105-2314-B-075A-003); and the Taichung Veterans General Hospital, Taiwan (TCVGH-1020101C, TCVGH-1020102D, TCVGH-1023102B, TCVGH-1023107D, TCVGH-1030101C, TCVGH-1030105D, TCVGH-1033503C, TCVGH-1033102B, TCVGH-1033108D, TCVGH-1040101C, TCVGH-1040102D, TCVGH-1043504C, and TCVGH-1043104B); it was also supported in part by the National Center for Advancing Translational Sciences (CTSI grant UL1TR001881).

T2D-Genes/GoT2D/DIAGRAM/SIGMA: J.C.F. is a Massachusetts General Hospital Research Scholar. M.I.McC. is a Wellcome Trust Senior Investigator. A.P.M. is a Wellcome Trust Senior Fellow under award WT098017. This work was supported by NIH grants DK062370, 1RC2DK0888389, U54HG003067, U01-DK085526, U01-DK085501, U01-DK085524, U01-DK085545, U01-DK085584, and by Wellcome Trust Grants 090532, 098381, 090367. The exome sequencing project in Mexican samples (SIGMA) was conducted as part of the Slim Initiative for Genomic Medicine, a project funded by the Fundación Carlos Slim in Mexico and partial funding for CONACyT grants 138826 and 128877.

HUNT study: The Nord-Trøndelag Health Study (The HUNT Study) is a collaboration between the HUNT Research Centre (Faculty of Medicine, Norwegian University of Science and Technology NTNU), the Nord-Trøndelag County Council, the Central Norway Health Authority and the Norwegian Institute of Public Health.

Malmo Diet and Cancer Study: This work was supported by grants from the European Research Council (consolidator grant 649021, Orho-Melander); Swedish Research Council (Orho-Melander and Melander); Swedish Heart and Lung Foundation (Orho-Melander and Melander); Knut and Alice Wallenberg Foundation-Clinical Scholar grant (Melander); Gustafsson Foundation (Melander).

United Kingdom Biobank: The authors would like to thank the UKBiobank and its participants for creating an open scientific resource for the research community. S.K. is supported by a Research Scholar award from the Massachusetts General Hospital, the Donovan Family Foundation, and R21HL120781.

Copenhagen Population Studies: The authors would like to thank the staff and participants of the Copenhagen General Population Study, the Copenhagen City Heart Study, and the Copenhagen Ischemic Heart Disease Study, collectively referred to as the Copenhagen General Population Studies.

Supplementary Tables

Supplementary Table 1. Clinical characteristics of 58,124 sequenced DiscovEHR study participants of European ancestry

**Age, year, median (IQR)**	61.3 (47.7-72.5)
Female sex, n (%)	34,549 (59.4%)
BMI, kg/m^2, median (IQR)	30.3 (26.1-35.7)
Fasting glucose, non-diabetic individuals, mg/dl, median (IQR)	94.0 (89.0-100.5)
Current smoker, n (%)	10,172 (17.5%)
**Medications**	
Lipid lowering medication, n (%)	21,898 (37.7%)
Anti-hypertensive medication, n (%)	27,272 (46.9%)
Hypoglycemic medication, n (%)	15,678 (27.0%)
**Medical history**	
Coronary artery disease, n (%)†	13,626 (23.4%)
Hypertension, n (%)‡	35,395 (60.9%)
Type 2 Diabetes, n (%)§	12,969 (22.3%)

†Participants were considered to have coronary artery disease if they had a history of coronary revascularization in the electronic health record, or history of acute coronary syndrome or exertional angina with angiographic evidence of obstructive coronary atherosclerosis. ‡Participants were considered to have hypertension if they had a history of hypertension in the electronic health record, antihypertensive medication use, or systolic blood pressure greater than 140 mmHg or diastolic blood pressure greater than 90 mmHg. §Participants were considered to have diabetes if they had at least 2 out of (i) a history of type 2 diabetes in the electronic health record, (ii) antidiabetic medication use, or (iii) fasting glucose greater than 126 mg/dl or hemoglobin A1c greater than 6.5%.

**Supplementary Table 2. Rare predicted loss of function variants in *ANGPTL4***				
**Chromosome**	**Position**	**Mutation**	**Mutation Type**	**Carriers**
19	8364556	p.Cys80fs	Frameshift indel	1
19	8364625	p.His103fs	Frameshift indel	1
19	8365994	p.His121fs	Frameshift indel	1
19	8366041	p.Arg136Ter	Nonsense	2
19	8366063	p.Gln143fs	Frameshift indel	2
19	8366280	p.Gln170Ter	Nonsense	6
19	8366320	c.547+1G>A	Splice donor	11
19	8369290	p.Gln207fs	Frameshift indel	1
19	8369320	p.Lys217Ter	Nonsense	1
19	8371116	p.Trp241Ter	Nonsense	1
19	8371240	c.758-1G>T	Splice acceptor	2
19	8371418	p.Gly313fs	Frameshift indel	69
19	8371437	p.Ser320fs	Frameshift indel	1
19	8373703	c.1040-2A>G	Splice acceptor	10
19	8373715	p.Trp350Ter	Nonsense	1
19	8373720	p.Thr353fs	Frameshift indel	1
19	8373744	p.Gln362fs	Frameshift indel	2
19	8373754	p.Tyr363Ter	Nonsense	3
19	8373770	p.Gln369Ter	Nonsense	6
19	8373826	p.Tyr387Ter	Nonsense	2
19	8373878	p.Ser406fs	Frameshift indel	1
Total				125

Supplementary Table 3. Age distribution of DiscovEHR participants					
**Cohort**	**Median (IQR) age, cases**	**Median (IQR) age, controls**	**Median (IQR) age, E40 homozygotes**	**Median (IQR) age, E40K heterozygotes**	**Median (IQR) age, K40 homozygotes**
DiscovEHR study	67.5 (18.4)	58.7 (26.7)	59.3 (25.6)	59.3 (25.8)	66.1 (23.9)
DiscovEHR replication cohort	63.6 (17.3)	51.8 (27.5)	61.7 (24.7)	61.3 (24.5)	66.7 (21.4)
Combined	63.6 (17.3)	51.8 (27.5)	54.1 (26.8)	54.4 (27.1)	61.6 (22.2)
Abbreviations: IQR, interquartile range.					

Supplementary Table 4. Studies for association of p.E40K with type 2 diabetes and glucose

Study	Ancestry	Allele frequency - rs116843064	T2D Case definition	T2D Cases	T2D Controls	Genotyping method
DiscovEHR Study	European American	0.02	At least 2 out of (i) a history of type 2 diabetes in the electronic health record, (ii) antidiabetic medication use, or (iii) fasting glucose greater than 126 mg/dl or hemoglobin A1c greater than 6.5%.	12,945	36,165	Whole exome sequencing
T2D-Genes/GoT2D /DIAGRAM	Multiple	0.0211	Multiple	34,809	57,985	Exome array+exome sequencing
Copenhagen General Population Studies	Danish European	0.0260	ICD code of type 2 diabetes (ICD-8 code 250; ICD-10 code E11; E13 or E14) on the basis of data in the national Danish Patient Registry or the national Danish Causes of Death Registry, self-reported diabetes, use of antidiabetic drugs, or a non-fasting plasma glucose concentration of greater than 11 mmol/l.	7,838	107,246	TaqMan-based assay, allele-specific PCR
deCODE	Icelandic	0.0243	Clinician-confirmed type 2 diabetes, or type 2 diabetes oral medication x 3 or more (or x 2 and current), or HbA1c x2 or more at least 6.5%, or HbA1c once at least 6.5% and oral medication (if information available) and either self-reported type 2 diabetes diagnoses or hospital discharge diagnoses of type 2 diabetes, and with confirmed type 1 diabetes diagnoses excluded.	6,808	72,309	HumanHap300, HumanCNV370, HumanHap610, HumanHap1M, HumanHap660, Omni-1, Omni 2.5 or Omni Express, whole genome sequencing+imputation
The HUNT Study	Norwegian European	0.0292	Persons with 1) an ICD9 (1987-1999) or ICD10 (1999-2016) code specific for type 2 diabetes obtained from local hospitals, 2) a non-fasting capillary or serum glucose measurement >11.1 mmol/L (200mg/dL) obtained from one of three HUNT health surveys (1984-1986, 1995-1997 and 2006-2008), or 3) a HbA1c value ≥ 6.5% (7.0 mmol/L) obtained from a subset of persons at one HUNT health survey (1995-1997). All other persons were considered as controls.	4,761	56,837	Exome array
Malmo Diet and Cancer Study	Swedish European	0.0282	Self report of physician diagnosis of diabetes or being on antidiabetic medication or having a fasting plasma glucose >7.0mmol/L or being registered in any of 6 different Swedish registers of diabetes diagnosis *(11).*	4,854	23,060	Exome array
UK Biobank	British European	0.0193	Report of diabetes unspecified or type 2 diabetes during verbal interview with trained nurse or current use of antidiabetic medication.	5,741	106,597	UK biobank array+UK BiLEVE array
EPIC InterAct Core-Exome	British European	0.0259	Report of type 2 diabetes from two or more of the following: self-report, linkage to primary care registers, secondary care registers, medication use (drug registers), hospital admissions and mortality data.	5,143	7,300	Core Exome array
DiscovEHR 30K replication cohort	European American	0.0214	At least 2 out of (i) a history of type 2 diabetes in the electronic health record, (ii) antidiabetic medication use, or (iii) fasting glucose greater than 126 mg/dl or hemoglobin A1c greater than 6.5%.	3,456	22,372	Whole exome sequencing
EPIC InterAct Quad660	British European	0.0250	Report of type 2 diabetes from two or more of the following: self-report, linkage to primary care registers, secondary care registers, medication use (drug registers), hospital admissions and mortality data.	4,257	4,293	Quad660 array+imputation
MGI	European American	0.0194	eMERGE *(1)*	1,389	12,289	Exome array
Duke	European American	0.0168	Clinical assessment by cardiology fellow at the time of caridac catheterization, based upon clinical information and judgement.	1,630	4,903	Whole exome sequencing
EPIC-Norfolk	British European	0.0179	Report of type 2 diabetes from: self-report, linkage to primary care registers, secondary care registers, medication use (drug registers), hospital admissions and mortality data.	1,347	19,504	UK Biobank array
UPenn	European American	0.0177	At least 2 out of (i) a history of type 2 diabetes in the electronic health record, (ii) antidiabetic medication use, or (iii) fasting glucose greater than 126 mg/dl or hemoglobin A1c greater than 6.5%.	733	4,066	Whole exome sequencing

Abbreviations: T2D, type 2 diabetes.

**Supplementary Table 5. Associations of p.E40K with fasting glucose**					
**Study**	**Scale**	**Beta**	**SE**	**P Value**	**N**
DiscovEHR	log10(mg/dl)	-0.001	0.001	0.38	26,644
DiscovEHR 30K replication	mg/dl	0.132	0.375	0.73	13,732
UPenn	log10(mg/dl)	-0.012	0.007	0.10	5,782
T2D-Genes/GoT2D/DIAGRAM	mmol/l	-0.021	0.011	0.06	33,245
Malmo Diet and Cancer Study	log10(mmol/l)	-0.009	0.057	0.87	4,848
The HUNT study	log10(mmol/l)	-0.0002	0.004	1.00	2,714
deCODE	Standard units	-0.005	0.0019	0.010	33,085
Meta-analysis				0.0024	120,050
Abbreviations: SE, standard error					

Supplementary Table 6. Associations of p.E40K with oral glucose tolerance test measures of glucose metabolism

Trait	N	Beta (Z score units)	SE	P Value
Glucose, 0 min (mmol/L)	8079	-0.0135	0.0465	0.77
Glucose, 30 min (mmol/L)	8081	-0.0293	0.0445	0.51
Glucose, 120 min (mmol/L)	8069	-0.0537	0.0459	0.24
Insulin, 0 min (mU/L))	8025	-0.0739	0.0431	0.086
Insulin, 30 min (mU/L)	7922	-0.1472	0.0459	0.0013
Insulin, 120 min (mU/L)	7899	-0.1068	0.0445	0.017
HOMA-IR	8023	-0.0719	0.0432	0.096
Insulinogenic index	7886	-0.1466	0.0474	0.002
Insulin sensitivity index	7912	0.1292	0.0430	0.0026
Disposition index	7886	-0.0370	0.0463	0.42

All the traits were rank inverse transformed and converted to Z score units. Association analyses were performed using linear mixed models of association adjusted for age, sex and body mass index. A genetic relatedness matrix was included in each model as a random-effects covariate. Abbreviations: HOMA-IR, homeostatic model assessment of insulin resistance; SE, standard error.

Supplementary Table 7. Studies for association of *ANGPTL4* predicted loss of function variants with type 2 diabetes

Study	Ancestry	T2D Case definition	T2D Cases	T2D Controls	Ascertainment of *ANGPTL4* pLoFs
DiscovEHR Study	European American	At least 2 out of (i) a history of type 2 diabetes in the electronic health record, (ii) antidiabetic medication use, or (iii) fasting glucose greater than 126 mg/dl or hemoglobin A1c greater than 6.5%.	12,969	36,217	Whole exome sequencing
DiscovEHR 30K replication cohort	European American	At least 2 out of (i) a history of type 2 diabetes in the electronic health record, (ii) antidiabetic medication use, or (iii) fasting glucose greater than 126 mg/dl or hemoglobin A1c greater than 6.5%.	3,456	22,372	Whole exome sequencing
UPenn	European American	At least 2 out of (i) a history of type 2 diabetes in the electronic health record, (ii) antidiabetic medication use, or (iii) fasting glucose greater than 126 mg/dl or hemoglobin A1c greater than 6.5%.	734	4,066	Whole exome sequencing
Duke	European American	Clinical assessment by cardiology fellow at the time of caridac catheterization, based upon clinical information and judgement.	1,630	4,903	Whole exome sequencing
TAICHI	Taiwanese Chinese	Fasting glucose > 126 mg/dl or 2 hour post-prandial glucose > 200 mg/dl, or on antidiabetic medication	4,392	4,699	Whole exome sequencing
T2D-Genes/GoT2D/SIGMA	Multiple	Multiple	8,373	8,466	Whole exome sequencing
Dallas Heart Study	Multiple	Fasting glucose > 126 mg/dl or on antidiabetic medication	461	3,283	Whole exome sequencing

Abbreviations: pLoFs, predicted loss of function variants

Supplementary Figures

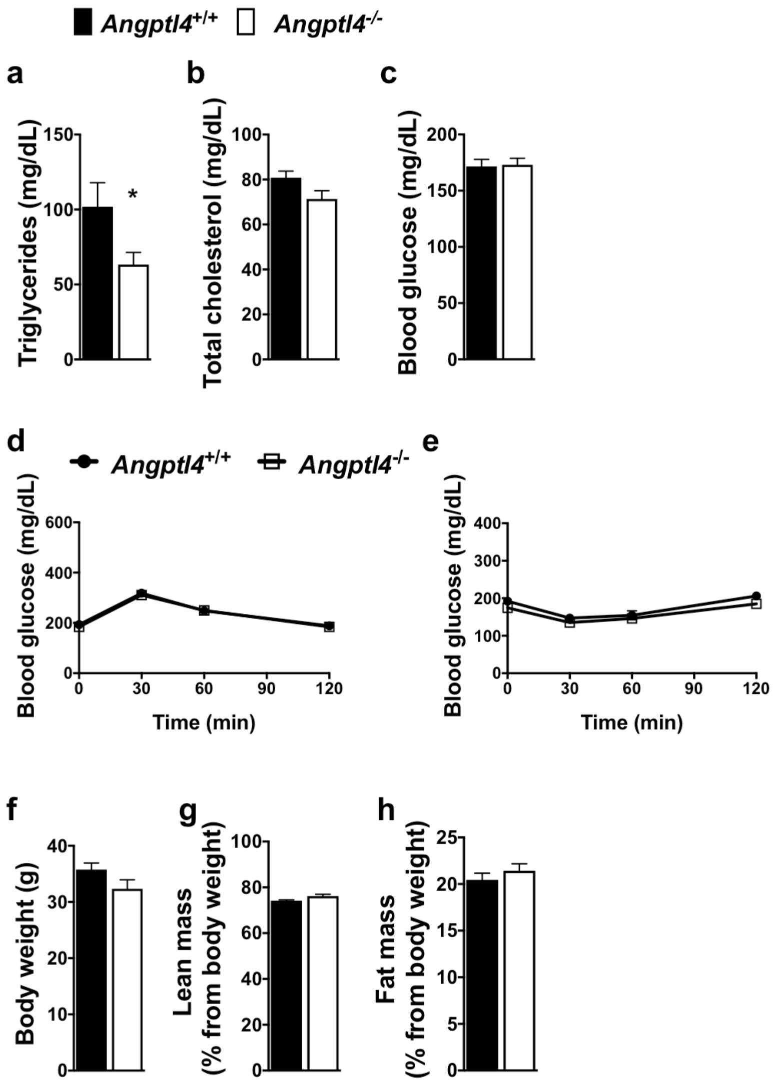

Supplementary Figure 1. Angptl4-/- mice on chow diet have reduced serum triglycerides and no change in glycemic control. (**a**) Serum triglycerides, (**b**) total cholesterol and (**c**) blood glucose levels in *Angptl4*$^{-/-}$ and control mice on chow diet. (**d**) Oral glucose tolerance test and (**e**) insulin tolerance test in the animals described in (**a-c**). (**f**) body weight, (**g**) lean and (**h**) fat mass in *Angptl4*$^{-/-}$ and their wildtype littermates. All groups had 9-11 animals. Values are mean ± SEM. Statistical analysis by Welch's t-test (**a**) and 2-way ANOVA with Sidak's post-test (**d** and **e**), *p < 0.05.

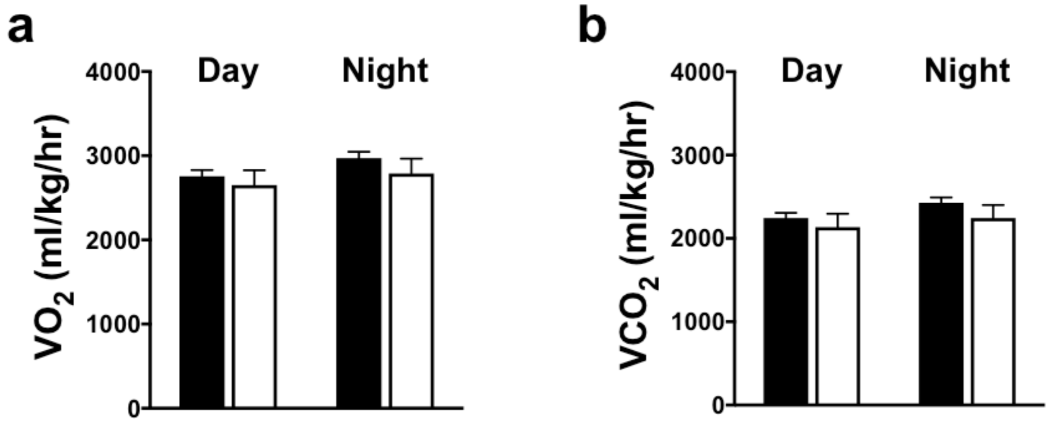

Supplementary Figure 2. Angptl4-/- mice on high-fat diet have no changes in O2 consumption and CO2 production. (**a**) O_2 consumption and (**b**) CO_2 production were evaluated during light and dark cycles in *Angptl4*$^{-/-}$ mice on high-fat diet**.** All values are mean ± SEM.
