# Supplementary material for: Genetic inactivation of ANGPTL4 improves glucose homeostasis and is associated with reduced risk of diabetes
Source: Nat Commun. 2018 Jun 13;9:2252. doi: 10.1038/s41467-018-04611-z (PMC5997992; doi:10.1038/s41467-018-04611-z)
Supplement: Supplementary file 2 — Description of Additional Supplementary Files [file 41467_2018_4611_MOESM2_ESM.pdf]

Description of Additional Supplementary Files

File Name: Supplementary Data 1
Description: Clinical characteristics of ANGPTL4 p.E40K homozygotes.

File Name: Supplementary Data 2
Description: Phenome-wide association analyses of p.E40K with disease diagnoses in DiscovEHR.

File Name: Supplementary Data 3
Description: Phenome-wide association analyses of p.E40K with clinical measurements in DiscovEHR
